# Behavioural risk factors for sexually transmitted infections and health seeking behaviour of street youths in Ibadan, Nigeria

**DOI:** 10.4314/ahs.v18i1.23

**Published:** 2018-03

**Authors:** Adeyimika Titilayo Desmennu, Musibau Ayoade Titiloye, Eme Theodora Owoaje

**Affiliations:** 1 Department of Health promotion and Education, FAculty of Public Health, University of Ibadan; 2 Department of Community Medicine, College of Medicine, university of Ibadan

**Keywords:** street youth, risky sexual behaviour, sexually transmitted infections, health seeking behaviour, risk reduction

## Abstract

**Background:**

Street youths are faced with a number of health challenges that could be linked to their exposure to the risk elements, accessing medical care including motivation and /or ignorance to utilise available health care.

**Objective:**

This qualitative study therefore aimed at determining the behavioural risks for sexually transmitted infections (STIs) and health seeking behaviour of street youths in Ibadan.

**Methods:**

Sixteen focus group discussion (FGD) sessions were conducted among 160 street youths aged between 15–24 years.

**Result:**

The result showed that most of the respondents had low perception of risk of contracting STIs in spite of their risky behaviours which included multiple sexual partnering, sharing of personal effects, malnourishment and sexual harassment. Most of the street youth could not identify the various types however; Gonorrhea and HIV were commonly mentioned by them. The major treatment regimen for STIs was traditional remedies and drugs obtained from patent medicine vendors. Traditional remedies were preferred by most of the participants and considered to be more effective.

**Conclusion:**

Majority of the street youths were sexually active, engaged in high risk sexual behaviours and had inappropriately treated sexually transmitted infections. Development of risk reduction and appropriate sexual health interventions targeted at prevention and appropriate treatment is recommended.

## Introduction

The phenomenon of homeless/street children is global, with the streets throughout the world being home to millions of children^1^,^2^. Tens of millions of children are living or working on the world's street. This number keeps growing due to population growth, intensifying urbanisation and migration, particularly in the developing world, amongst others^3^.

Children are pushed into living and working on the street by many factors such as Natural disasters, wars, poverty, economic recessions, domestic abuse and family crises, or even the ideal of freedom that is thought to be found on the streets are all among the reasons causing boys and girls, young men and women to seek means for survival^2^,^4^, and many times, they take to street life in order to relieve parents of burden of care^5^. Once on the streets many other threats await these children; some of the most pressing challenges street children face include difficulties in maintaining basic health and accessing health services, violence and abuse, and dangerous working conditions which makes them vulnerable to various kinds of psychological problems and health risks^3^,^6^.

Basic needs for money, food, shelter and other essential necessities of life co-join with early initiation of sexual activity^7^ place street children at high risk of becoming involved in the sex trade or of practicing ‘survival sex’ in exchange for food, shelter, money or drugs^8^,^9^. Substance use also increases the likelihood that individuals will engage in risky sexual behaviours, for example, non-condom use and multiple sexual partner patterns which put them at risk of sexually transmitted infections (STIs) and HIV/AIDS^10^,^11^,^6^.

In spite of the visibility of the street youths, they have limited access to basic services, such as education, healthcare and housing drawing attention to them for advocacy and policy-making by the government4. Street children reported limited access to health care. Barriers^12^ included cost, minority status, stigmatization by providers, distrust in quality of care, and difficulty finding time to seek care because of lost or meagre earnings^13^,^14^. Two studies found that a majority of street youth did not seek medical help for ailments instead they tend to ignore their symptoms or self-medicate when ill^12^.

Evidence from studies in sub-Saharan countries indicate that many young people on the streets are sexually active and engage in risky sexual behaviours such as unprotected sexual intercourse, commercial sex, homosexuality and multiple sexual partnering^6^,^15^,^16^. Araoye^17^ in a study of female adolescent hawkers in motor parks and truck stops in Ilorin, Nigeria reported that half of the respondents were sexually experienced and their first partner was someone within the motor park. Sixty percent claimed to have been sexually harassed by men while hawking and the harassment was usually accompanied by promises of gifts. In another study by Olley^18^ emphasis was laid on street life and participation in the street culture which often increases the vulnerability of street youths to sexual health problems including STIs among other possibilities.

The hawkers were also exposed to sexual exploitation, harassment, coercion, and abuse. Asante^6^, in a study of street children in Accra, Ghana; reported alcohol use as independently associated with the four indices of risky sexual behaviour (ever had sex, non-condom use, multiple sexual partners and survival sex). In South Africa, Swart-Kruger and Richter^19^ reported that the street children were knowledgeable about HIV/AIDS but their perceived risk was low.

## Rationale

Research indicates that out of school youths in Nigeria are generally more sexually active and participate more frequently in risky sexual behaviours than their counterparts in school^20^. Practices such as networks of concurrent sexual relationships which have been implicated as the primary cause of the spread of HIV in parts of sub-Saharan Africa^21^ are also reportedly more common in youths on the streets (Dare, Oladepo, Cleland and Badru, 2001). Increased use of illicit drugs as a coping mechanism with the daily stresses had also been reported among these youths. A study of street youths in South Western Nigeria reported that 30% used alcohol and other drugs such as tobacco (14%) and cannabis (10%)^22^. Drug use among street youths has been associated with unprotected sex^23^. The risky sexual behaviours among this population and other behaviours such as drug use are of major public health and social health concern, but there is paucity of current information on these behaviors and the reproductive health needs of these young people. Though the number of young people on the streets has been on the increase in the urban areas of the country, most reproductive health programs have not addressed the reproductive health needs of this vulnerable group. Therefore, the objective of this research was to investigate the risky sexual practices and health seeking behaviours of young people on the streets around the markets and motor parks in Ibadan with the view of developing appropriate interventions to reduce the risk of contracting STIs including HIV and also to educate them on appropriate treatment regimen in case of ill-health.

## Methodology

### Study design

The study was a qualitative one based within the phenomenological paradigm which attempts to understand people's perceptions, perspectives and understandings of a particular situation. This approach was chosen because of its usefulness in exploration of people's knowledge views and experiences. The other advantage of qualitative methods is that they can be participatory, democratic and empowering^24^. The Focus Group Discussion (FGD) method was used to collect information from study participants utilising an FGD guide which comprises questions related to the background and street life of the participants (including reasons for being on the street), health problems they are faced with, knowledge of sexually transmitted infections including their perceived susceptibility and risks. Additionally, questions related to drug use and abuse, exposure to risks, ailments and treatment options adopted by them when they take ill were asked during the sessions. The discussion sessions were facilitated by the investigator in the indigenous language of the participants, which is Yoruba. This descriptive study carried out in major markets, motor parks and commercial areas in Ibadan North and Ibadan South East Local Government Areas (LGA) i.e. Bodija, Mokola, Gate and Oranyan/Bere/ Mapo were the study sites. These areas are a beehive of activity where young people are often found till late in the night. Sixteen focus group discussions were carried out in selected markets and motor parks in Ibadan. This included Bodija and Mokola, Gate and Oranyan respectively. Four FGDs: two female and two male groups were conducted in each of the selected study areas.

### Study area

The study location was Ibadan, the capital of Oyo State, which is one of the 36 states in Nigeria. Located in the South-Western part of Nigeria, 90 miles North of Lagos, the city consists of five urban local government areas and has an estimated population of 3 million people approximately 700,000 are under 16 years^25^ Ibadan, which is an old city, constitutes many tribes, but is dominated by Yoruba-speaking people.

### Study population and sampling

The study population consisted of young people aged between 15 and 24 years in street circumstances i.e. involved in daily street-based activities including unskilled labor, which included scavenging, motor-park tout, bus boy/vehicle conductors, load carriers/head loading (these are structured groups in the study areas — motor parks and markets) or wandering about on the streets. The study participants in the study areas were identified in groups based on the activity they were engaged. These groups were identified by the research team with the assistance of the community mobilisation officers and by peer recruitment in the two local government areas. Due to the scope of the study, children who were less than 15 years and youths older than 24 years, those who normally returned to a family at the end of the day and those who were less than one year on the street were excluded from the study. Youth between the ages of 15 — 19 years constituted the younger group while those between the ages of 20 – 24 years constituted the older youth group and an average of 10 persons were in each discussion group. FGD sessions were held separately by gender and age group of the participants. An extensive community mobilization strategy was utilized in recruitment of the participants. Monetary incentives were not encouraged therefore a consensus was reached on the use of towels and refreshment in the form of fruit drinks and biscuits which were distributed during each session of the FGD.

### Ethical considerations

Prior to the commencement of the research, ethical approval to conduct the research was obtained from the Oyo State Ethical Review Committee after an in-depth review of the proposal for compliance with ethical guidelines. Also, permission was obtained from the leaders of the identified groups where necessary. The criteria for selection of samples included voluntary declaration for participation in the study which was documented through an informed consent form, and the ability for transmission of information^26^ respect for persons, especially since the research had no invasive components and posed very minimal risk to the participants. Especially since the parents or guardian were usually out of reach. Permission was also sought from the participants for the use of a tape recorder during the discussion session and this would facilitate capturing of salient information which would come up from the FGD sessions.

### Data collection and analysis

Information collected among the subjects were transcribed verbatim, word processed and thematically analyzed.

## Results

The following themes were identified from the focus group data and are reported below: (i) Perception of the concept of street youth and health problems. (ii) Knowledge of and perceived susceptibility to sexually transmitted infections (STIs). (iii) Types of treatment for STIs. (iv) Suggested interventions to reduce the spread of STIs.

### Perception of the concept of street youth and health problems.

Street youths were regarded by majority of the discussants as young people, either male or females but mostly males who are poor, jobless, homeless, runaway youths who sleep under bridges in garages and other make shift shelters. They are mostly from dysfunctional families and usually have to fend for themselves Most of these youth smoke cigarettes and marijuana; drink alcohol, engage in drugs and are involved in other dangerous behaviours including sex work. They usually lack care, they could be orphans but most of them have parents. As said by one of the male discussants;

“*There is no one to take care of us and provide what we need, so we have to come and work to meet our needs”.*

Another said; “even when our parent or guardian are around, we still have to work to support the home”

Street youths are said to work mostly as bus conductors, truck pushers, hawkers/petty trading and are involved in a lot of crime.

- “ *we have to do whatever we find around , we have to survive so we don't have a choice and we get roped into a lot of crime*”

Health problems mentioned by the participants include malaria, headaches, respiratory infections, skin diseases, diarrhoea, dysentery and injuries which usually resultsfrom their dangerous way of life. Majority of the females are reported to be exposed to rape, unwanted pregnancies and ritual kidnap. They generally faced embarrassment linked with false accusation including imprisonment and sometimes death. They are usually prime suspects of any criminal act. “We fall sick very often, we have headache, stomach pain, stooling……..”

“*We also have lots of injuries when we work ……….*”

“*Some of our friends get lost, we just wake up and do not find them, and we later hear that they have been stolen (kidnapped)*”

Other problems encountered by street youth include; accidents, substance use, heart problems, hypertension which can result from smoking cigarette, marijuana and cocaine which as stated by a participant “*can also result into madness*”, unwanted pregnancies and abortion with all its complications including death. Ritual killing (when a person is killed for sacrificial purposes), rape, kidnap, lack of / poor access to formal education, ill health, poor nutrition, misuse of drugs which could lead to many other complications were some other problems mentioned by the discussants. These can be categorised into violence and sexual abuse — kidnap, ritual killing, rape; risky behaviours - unsafe and unprotected sex, unwanted; adolescent reproductive health issues — unwanted pregnancy, abortion; drug use/abuse — smoking cigarette, marijuana, cocaine. Others include general health challenges — hypertension, poor nutrition etc.

### Knowledge of and perceived susceptibility to sexually transmitted infections (STIs)

Concerning their knowledge about STIs, majority of the discussants had heard about STIs although, they could not identify the various types. The most commonly mentioned was Gonorrhea, while a few mentioned Syphilis. However, some of them wrongly believed that Diabetes, yellow fever and malaria can be transmitted through sex. They knew that there were other types of STIs by observed signs and symptoms such as ulcers around the genital area, offensive discharges from the genitals, pain during urination; but could not identify the differences. Their most common source of information about STI was the mass media i.e. television and radio, while a few heard through interpersonal discussion with friends.

About half of the discussants identified HIV/AIDS as a STI, but most knew that it is mostly transmitted through sex. Majority of the discussants affirmed that STIs and HIV/AIDS are diseases that constitute a major threat to street youth. The participants were aware that use of condoms is a sure way to reduce and most likely prevent STI infections although their reported use is low due to reasons like reduction in sexual satisfaction with use of condoms.

Most of the discussants believed that street youth especially females are susceptible to contacting STIs which includes HIV. This is due to the fact that many of the female street youth could easily be lured, forced or coerced into sexual intercourse against their will because they lack required physical, emotional, social and financial empowerment to enable them negotiate sex. Also, many of the female youths on the street are of the younger age group i.e. between 14 and 19 years. These younger age groups are usually taken advantage of by the older men that they sell goods to or just hang out with.

However, when asked personally whether they are susceptible to contracting STIs, almost all the males said “no”. When enquiries were made about what they do to protect themselves, they were not willing to discuss this; they felt, the means of protecting themselves was “their little secret of life”. While the males were outspoken about sexuality issues, the females were generally quiet and uncertain in their answers.

### Types of treatment for STIs

The two major ways that youth treat these infections are through the use of traditional medicine and modern methods of treatment. Majority of the youths reported that they do not go to hospitals but they visit Patent Medicine Vendors (PMV — informal outlets to over the counter drugs) to obtain drugs without formal prescription. These drugs are usually taken together with the readily available local remedies containing alcohol and traditional medicines.

“*We use a number of medicine at the end of the day when we are tired and when we are sick……………………*.”

“*Some of these drugs are given to use by those who have been using them and found out it works for them*”.

“*The materials we mix are our own personal secret*…

Majority of the youths preferred the adoption of traditional methods of treatment although, about half of them believed that the two methods could be combined. Only a few of the discussants said modern medicine does not cure the diseases it only suppresses the pain for a while. As said by one of them “*traditional methods works and cures faster, they are very effective*”. Examples of concoctions used include; a mixture of Guinness beer, palm-wine and mango tree bark soaked together for a day after which it can be taken, mixture of herbs with hot drinks, “*dongoyaro*”, “*sepe*” and unripe pawpaw.

### Suggested interventions to reduce the spread of STIs

Some intervention activities suggested by the youths for prevention the spread of HIV and AIDS including other STIs were “proper (strategic) enlightenment programmes, group talks, organization of programmes that will encourage the use of condoms and modern medicine, promotion of abstinence, provision of jobs and sponsorship for those who want to go to school or continue schooling by the government, provision of good quality latex condoms (like rough riders — softer than the others) in large quantity and provision of more free HIV test centers, general care for the street youths and provision of housing scheme for the homeless youths”. Some of the discussants expressed their concerns as follows:

“*let the government provide good jobs for us that we can do*”

“*I will like to still go to school, I will be glad if I get someone to help me*”

“*we are human too, so if they give us the condoms, like rough riders we will play safe. Rough riders have different types; some next to skin, some are embossed with patterns / studded to help us enjoy sex more*”…

“*we can also form groups to help ourselves, especially the younger ones*”

Youth can be involved in interventions to improve their sexual and reproductive health in a number of ways. These are:

1. Formation / organization of youth clubs (i. e. support groups)
2. Organization of care and counselling groups - to take care of employment for youth, while working together with the government.
3. Utilization of peer education as a strategy for behaviour change among youths

## Discussion

Significant findings from this study were that; the challenges and problems faced by street youths are varied and depend on their gender, majority of the discussants had heard about STIs but with wrong beliefs about their causes and mode of transmission. The two major ways that youth treat these infections are through the use of traditional medicine and modern methods of treatment. Intervention activities were suggested to reduce the spread of STIs among street youth.

Similar to reports from Columbia^27^ and Pakistan^28^ poverty was cited as the main reason for their being on the street. The day-to-day life of the youth was characterized by a constant struggle to generate income and obtain sufficient food. This plight was frequently compounded by different health problems that are related to the unhygienic surrounding and exposure to adverse conditions due to their quest for survival^28^,^29^.

In partial conformity to the study carried out in Pakistan by Ali and de Muynck,^28^ in which common illnesses mentioned by street children were persistent high-grade fever, injury, severe diarrhoea and hepatitis (yellow skin), Health challenges of street youths in SouthWest Nigeria included malaria, headaches, respiratory infections, skin diseases, diarrhoea, dysentery and injuries.

The majority of the participants had some knowledge of STIs although, they inadequately described the symptoms, signs and manifestations of the infections; many could not associate the different STIs with their signs and mode of transmission. However, majority of the participants had some knowledge of HIV, its mode of transmission and prevention. This is consistent with the study on HIV knowledge and sexual risk behaviours of street children in Takoradi, Ghana; however, they reported believing that they were not susceptible to contracting HIV^30^. According to Tyler et al^31^ female youth on the street are more likely to have STIs compared with males. This is confirmed in this study in that female street youth were considered to be more at risk than their male counterpart. This is could be due to the fact that many of the female street youth could easily be lured, forced or coerced into sexual intercourse against their will because they lack required physical, emotional, social and financial empowerment to enable them negotiate sex. Also, many of the female youths on the street are of the younger age group i.e. between 14 and 19 years. These younger age groups are usually taken advantage of by the older men. Also, females tend to have less control over the situation when it comes to condom negotiation and use^32^.

The participants were aware that use of condoms is a sure way to reduce and most likely prevent STI infection although their reported use is low due to reasons like reduction in sexual satisfaction with use of condoms.

In accordance with some studies in Ghana and Pakistan (Wutoh et al 2006, Ali and de Muynck, 2005) majority of the street youth practiced self-medication and preferred the traditional health care to the medical/orthodox care. Traditional healers were economical and more effective, using safe herbal medicine. They have the reputation of knowing secret cures for chronic and incurable diseases, so they are the preferred choice in situations where modern medicine failed or financial constraints becomes a key factor (Ali and de Muynck, 2005).

## Conclusion

Findings from this study revealed that street youth in Ibadan metropolitan area are confronted with a number of health-related problems which directly or indirectly have negative impact upon their livelihood. They unknowing are at great risk of contracting sexually transmitted infections due largely to the fact that the circumstances they often find themselves and their lifestyle is characterised by extreme deprivation especially regarding basic amenities, exposure to violence and violation of their rights. These leaves them with limited life choices and engagement in risky behaviours. Females are more at risk/ vulnerable to contracting STIs than males. Also, these street youths were likely to engage in self-medication and are liable to use traditional treatment than medical or orthodox treatment for any disease they contract. This has implication for control of sexually transmitted diseases among them.

Intervention efforts and activities suggested to bring about improved sexual health includes; organization of care groups - to take care of employment for youth, working together with the government, youth counseling other youths — utilizing the peer education method, formation / organization of youth clubs.
